# Ovarian cancer in a former asbestos textile factory worker: a case report

**DOI:** 10.1186/s40557-018-0277-1

**Published:** 2018-11-16

**Authors:** Sunwook Park, Jaechan Park, Eunsoo Lee, Huisu Eom, Mu Young Shin, Jungwon Kim, Dongmug Kang, Sanggil Lee

**Affiliations:** 1 Department of Occupational and Environmental Medicine, Kosin University College of Medicine, Busan, Republic of Korea; 2 Occupational Safety and Health Research Institute, KOSHA, 400, Jongga-ro, Jung-gu, Ulsan, 44429 Republic of Korea; 3 Department of Occupational and Environmental Medicine, Pusan National University, Yangsan Hospital, Yangsan, South Korea

**Keywords:** Ovarian neoplasm, Asbestos, Occupational diseases, Textile industry, Spinning work

## Abstract

**Background:**

The International Agency for Research on Cancer (IARC) defined that asbestos is a group 1 substance that causes lung cancer, mesothelioma (pleura and peritoneum), laryngeal cancer, and ovarian cancer in humans. Many studies on lung cancer, and mesothelioma caused by asbestos exposure have been conducted, but there was no case report of ovarian cancer due to asbestos exposure in Korea. We describe a case of ovarian cancer caused by asbestos exposure in a worker who worked at an asbestos textile factory for 3 years and 7 months in the late 1970s.

**Case presentation:**

A 57-year-old woman visited the hospital because she had difficulty urinating. Ovarian cancer was suspected in radiologic examination, and exploratory laparotomy was performed. She was diagnosed with epithelial ovarian cancer. The patient did not undergo postoperative chemotherapy and recovered. She joined the asbestos factory in March 1976 and engaged in asbestos textile twisting and spinning for 1 year, 2 years and 7 months respectively. In addition, she lived near the asbestos factory for more than 20 years. There was no other specificity or family history.

**Conclusion:**

Considering the patient’s occupational and environmental history, it is estimated that she had been exposed to asbestos significantly, so we determined that ovarian cancer in the patient is highly correlated with the occupational exposure of asbestos and environmental exposure is a possible cause as well. Social devices are needed to prevent further exposure to asbestos. It is also necessary to recognize that ovarian cancer can occur in workers who have previously been exposed to asbestos, and the education and social compensation for those workers are needed.

## Background

Ovarian cancer is one of the most fatal diseases among gynecologic tumors [1]. Ovarian cancer is divided into five major histological subtypes. Of these, epithelial ovarian cancer is the most common, which accounts for 90% or more of ovarian cancers occurring in advanced countries [2]. According to the annual report of cancer statistics in Korea in 2015 released by the National Cancer Center, there were 214,701 cases of cancer. Among them, 2443 ovarian cancers accounted for 1.1% of all cancers and 2.4% of all cases of female cancers. The incidence rate per 100,000 people was 9.6. By age, 23.7% were in their 70s and over, 23.3% in their 60s and 50s, and 16.6% in their 40s [3]. Although causes of ovarian cancer are not identified accurately, there are several common risk factors. Family history of ovarian cancer or breast cancer, mutation and abnormality of BRCA1 or BRCA2 gene, increasing number of ovulation, hormone replacement therapy, and old age increase the risk of ovarian cancer, whereas pregnancy, breast-feeding, use of an oral contraceptive reduce the risk [2, 4].

In many previous studies, asbestos was mentioned as a potential risk factor of ovarian cancer [5–8]. However, this was not widely recognized, and in March, 2009 the causality was specifically discussed at the International Agency for Research on Cancer (IARC) Monographs Working Group. There was a counterargument that previous studies on the causal relationship between asbestos and ovarian cancer had a small number of ovarian cancer cases and might have misclassify peritoneal mesothelioma as ovarian cancer [9]. IARC concluded that the development of immunochemical diagnostic techniques would have reduced this possibility, and that the causality between occupational exposure to asbestos and ovarian cancer had sufficient evidence even after considering all other types of bias as well as coincidence and confound [10, 11]. To assess the IARC’s conclusion of causality between asbestos and ovarian cancer in a quantitative manner, a meta-analysis was carried out targeting the cohort studies performed from 1982 to 2009. As a result, pooled SMR of ovarian cancer was confirmed to be 1.77 (95% CI: 1.37–2.28). This data involves cohort data mentioned by the IARC as well as unpublished data [12]. Through recently published studies, it was confirmed that in a study comparing 5741 female workers exposed to asbestos until 2010, the mortality rate of ovarian cancer significantly increased (SMR = 1.38, *p* < 0.05) [13]. In addition, a cohort study targeted at 1818 workers at an asbestos cement factory in Italy also found that SMR of ovarian cancer was 3.64 (95% CI: 0.99–9.33) [14]. In the 2012 report, the IARC concluded in an epidemiologic study targeted at humans that asbestos has a causal relationship with laryngeal and ovarian cancer based on sufficient evidences and also has a limited correlation with colorectal, rectal, pharynx, and gastric cancer [15].

The asbestos textile industry uses asbestos as a raw material to produce asbestos fibers, cloth, tape and gloves [16]. Since the industry was at the initial stage of using asbestos as a raw material, workers were exposed to a large quantity of asbestos during the production process [17], and the asbestos textile industry showed the biggest exposure to asbestos among asbestos-related industries [15, 17–20]. As for studies on the cancer mortality the asbestos textile industry, a cohort study targeted at 631 female workers compensated for asbestosis from 1979 to 1997 in Italy showed that among female workers in the asbestos textile industry, ovarian cancer occurred in a total of four workers and SMR was 5.26 (95% CI: 1.43–13.47) [21]. In a cohort study targeted at 889 male workers and 1077 female workers in the asbestos textile industry from 1946 to 1984, it was confirmed that a total of five workers died of ovarian cancer and SMR was 2.61 (95% CI: 0.85–6.09) [22]. A follow-up observational study that monitors, until 2013, 1083 female workers in the asbestos textile industry, who were exposed to a high concentration of asbestos (100 fibers/mL) in a short period of time, found that SMR of ovarian cancer was 3.03 (95% CI: 1.69–4.99) [23]. Such epidemiologic studies suggest a high causality between exposure to asbestos in the asbestos textile industry and onset of ovarian cancer.

Asbestos may be exposed through various routes related to environmental causes besides occupational reasons [24]. The first route is the case that workers in asbestos-related industries bring their working clothes home and thus, exposing asbestos to their family members. Although levels of exposure to asbestos in family members are unknown, it was reported that the concentration of asbestos in the house of a miner in South Africa was measured to be 2–11 fiber/L [25–27]. The second route is the case that asbestos from asbestos factory or mine is scattered in the air and spreads with the wind or is exposed when transporting asbestos materials via railroad or road. In overseas studies on the concentration of asbestos in the surrounding area of asbestos mine, concentration of asbestos in the surrounding area was reported to be 2.5 fiber/L in Italy and 1–17 fiber/L in France. As for Canada, it was reported to be 46 fiber/L in 1974 and 10 fiber/L in 1984 [25–27]. According to overseas studies related to the concentration of asbestos in the surrounding area of asbestos factory, concentration of asbestos was reported to be 0.6–2.2 fiber/L in the US and 7.8 fiber/L in Canada. As for Germany, in the places where 300 m, 700 m, and 1000 m away from the factory in the direction of wind, it was reported to be 2.0, 0.8, and 0.6 fiber/L, respectively [25, 27–29]. A study conducted in Japan found that malignant mesothelioma was 9.5 times more likely to be developed within a 500 m of asbestos factory and the degree of risk increased up to 2.5 km according to the direction of wind [30]. In a study measuring concentration of asbestos in the surrounding area of asbestos textile factory in Indonesia, it was confirmed that concentration of asbestos was low depending on the distance from the factory and since the distribution was equivalent to the direction of wind, asbestos was exposed from the inside of asbestos textile factory to the outside [31]. The third route is the case that asbestos contained in products or construction materials including asbestos is exposed. According to the studies on concentration of asbestos within overseas schools and buildings, concentration of asbestos was reported to be 1–40 fiber/L in the US, 0.5 fiber/L in the UK, 22 fiber/L in Austria, and 0.42 fiber/L in Canada [39, 32, 33].

Despite the conclusion of the IARC regarding causal relationship between asbestos and ovarian cancer, the number of asbestos-related ovarian cancer cases is few in overseas countries, and the cases have not been reported in Korea. In particular, there were no studies evaluating both occupational and environmental exposure to asbestos. Therefore, this study aims to report the ovarian cancer occurring in a worker who worked at an asbestos textile factory in the late 1970s by analyzing occupational and environmental exposure.

## Case presentation

### Patient information

Fifty-seven-year-old woman.

BMI: 27 kg/m^2^ (155 cm, 65 kg).

### Chief complaints

Difficulty in urination.

### Present illness

**Fig. 1:**
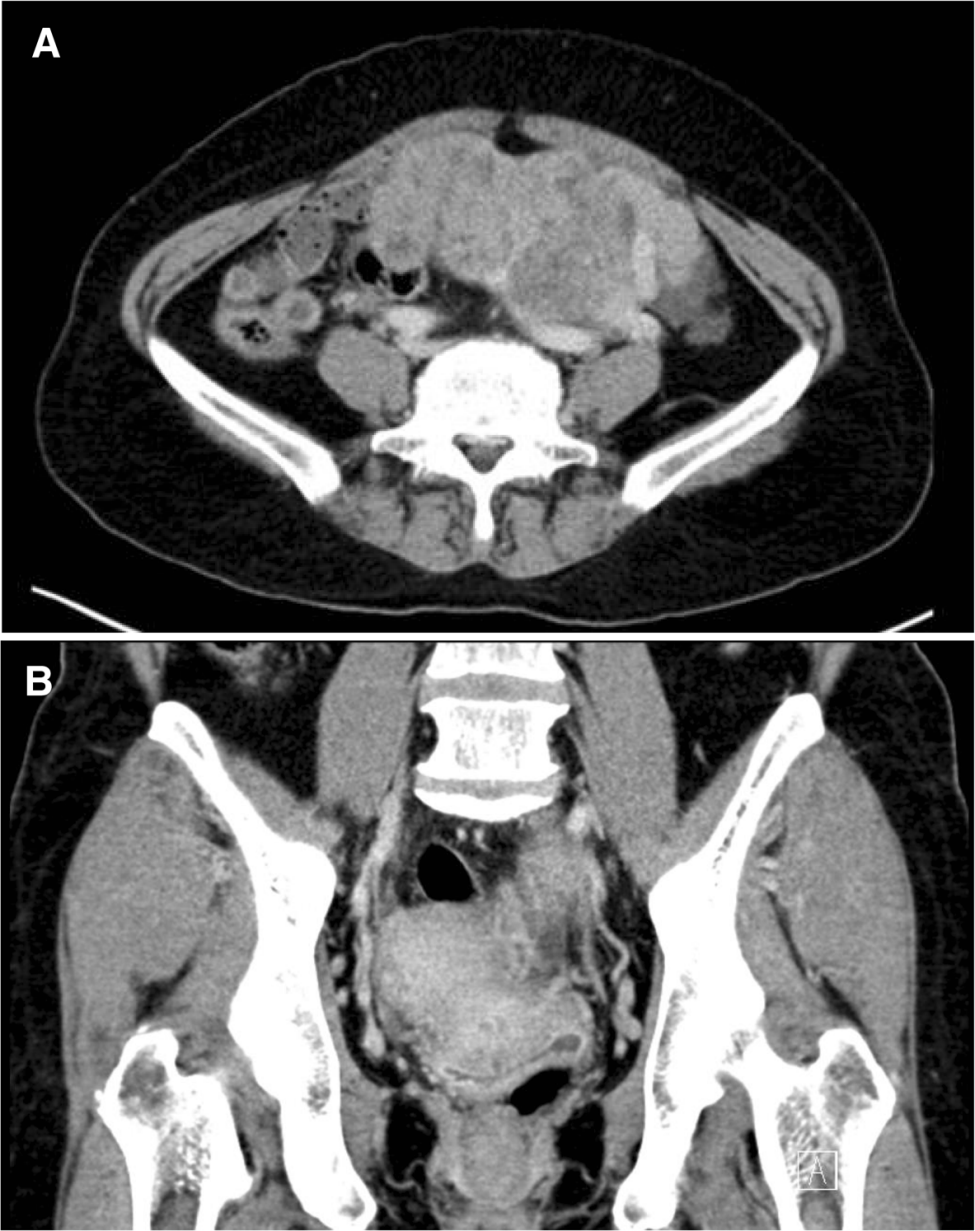
Computed tomography (CT) images showed lobulated soft tissue density mass (9.0 × 13.6 cm) suggesting left ovarian cancer. (**a**) axial image. (**b**) coronal image

**Fig. 2:**
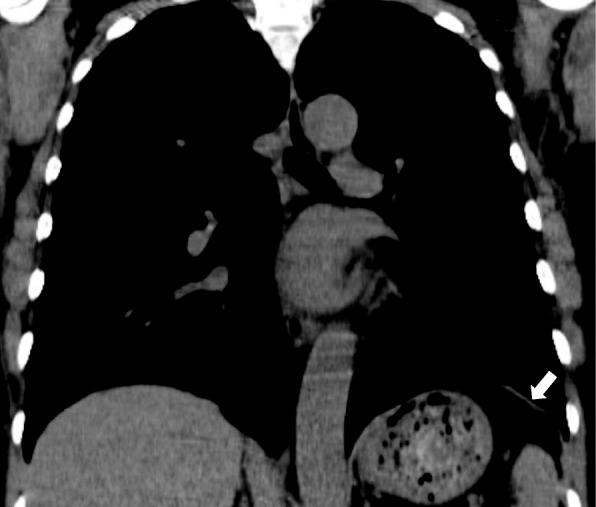
Coronal image with non-contrast chest CT scan shows a focal non-calcified plaque on the left hemidiaphragm

The patient suffered from a symptom having difficulty in urination from June, 2016. When she visited a local gynecology outpatient clinic due to abdominal discomfort on July 12, 2016, a huge uterine mass (heterogeneous 12 cm) was detected on the left through an ultrasound. Then, she was transferred to the department of Gynecology of Pusan National University Yangsan Hospital. A blood test conducted on July 13, 2016 showed that CA125 was 2543.1 U/mL, HE4 was 1361.6 pmol/L, and ROMA (postmenopausal) was 99.4261. In a subsequently conducted genetic test, BRCA 1, 2 turned out to be negative. A Pelvis CT conducted on July 15, 2016 showed a finding of suspected primary ovarian cancer (Fig. 1). Then, she underwent exploratory laparotomy (TAH, BSO, BPLND, PALND, Appendectomy, Omentectomy) on July 25, 2016. Finally, she was diagnosed with ‘ovarian cancer, serous carcinoma 1C, grade 1’ through a frozen biopsy. The patient refused to receive postoperative anticancer treatments and her condition has been monitored at the hospital outpatient department without any findings of a recurrence of the illness. On June 5, 2017, she visited the Occupational and Environmental Medicine outpatient clinic of Pusan National University Yangsan Hospital for work-relevance evaluation. At that time, she also had as symptom difficulty in breathing, accompanied with dry cough. Pulmonary function tests and chest X-ray showed no specific findings but through a chest CT conducted on June 20, 2017, she was diagnosed with suspicious asbestosis along with a finding of pleural plaque (Fig. 2).

### Past medical & obstetrical history

Parity 4–2–2-2. She married at the age of 23 and became the first full-term pregnant woman at the age of 25. All of her children were born with virginal delivery. She has breastfed all children for over a year. The patient underwent a sterilization operation when she was 29 years old. and reached menopause when she was 55 years old. She did not take any hormone replacement therapy after menopause. There was no special cancer history. A Pap smear test conducted in June, 2016 turned out to be negative.

### Social history & family history

Non-smoker, social drinker (3 standard drinks per week). No issues were found in the family history and medication history. Her husband worked for about 4 years as an automobile parts production worker.

### Occupational history

The patient performed chrysotile twisting and spinning works for one year and two years and seven months, respectively, at an asbestos textile factory from March, 1976 to October, 1979. She was sometimes sent to crocidolite part 1–2 times a month short for 2–3 days, long for 1 week, and usually worked a double shift day and nights for six days a week (08:00–19:00 in the daytime, 19:00–08:00 in the night time) but when there were a lot of work load, she worked for even seven days a week. At that time, workplaces were furnished with an air exhauster and workers wore a dust mask, but the levels of asbestos dust were very high since asbestos fibers easily broke into small particles during the process of spinning. Indeed, a thick layer of white dust sat on the scalp of workers even if they were hooded. As for working clothes, she washed them at home after work. She left the asbestos factory after marriage and then has been engaged in the restaurant business.

### Residential history

The asbestos factory where the patient worked was operated from 1969 to 1992 [34]. She lived around 1 km away from the factory before 1973, around 500–1000 m away from 1973 to 1982, and around 3.5 km away from 1982 until now.

### Exposure assessment

#### Occupational exposure

Since the asbestos factory no longer exists, levels of exposure to asbestos at the asbestos textile factory at that time were estimated through a literature review [15, 17–20]. Now that records on levels of exposure to asbestos in the 1970s when the patient worked are hardly left, the review was focused on the exposure to asbestos during the period most similar to her working period, among previous literature on levels of exposure to asbestos in the past. Among several asbestos textile process steps, levels of exposure during twisting and spinning process steps, in which the patient was involved, were estimated and the exposure values estimated through literature were considered to be the minimum exposure levels of the worker. The maximum exposure concentration based on Korean measurement data was confirmed to be 45.8 fiber/cc, which was measured during the weaving process at an asbestos textile factory in 1987. The levels of exposure to asbestos in the air at an asbestos textile factory from 1984 to 1992 are as shown in Table 1 [16–19, 35]. With regard to exposure levels by year, the earlier the time was, the higher the exposure level was. The weighted mean of exposure levels at the measured workplaces was confirmed to be 3.58 fiber/cc. Levels of exposure to asbestos showed a big difference depending on process step and type of works even within the asbestos textile industry. According to the data released by the Korea Occupational Safety & Health Agency (KOSHA) in 2006, exposure levels by process step in the asbestos textile industry are as shown in Table 2 [35]. If the maximum exposure levels during twisting and spinning process steps (14.9 and 15.0 fiber/cc, respectively) are applied to the patient, levels of exposure to asbestos are 14.9 fiber·year/cc (14.9 fiber/cc × 1 year) and 38.7 fiber·year/cc (15.0 fiber/cc × 2.58 year), respectively. If the geometric mean of exposure levels in 1987, most similar to her working period, is applied, levels of exposure to asbestos are 4.8 fiber·year/cc (4.8 fiber/cc × 1 year) and 14.45 fiber·year/cc (5.6 fiber/cc × 2.58 year), respectively. In conclusion, it is presumed that the patient was exposed to 19.25 or 53.6 fiber·year/cc of asbestos or more while working at an asbestos textile factory for three years and seven months.

**Table 1:** Asbestos exposure levels in the air at asbestos textile factories in Korea (1984–1992)

Period	Level of exposure (geometric mean, fiber/cc)	Range of exposure (fiber/cc)	Number of measurement workplaces	Reference
1984	6.28	0.62–30.73	6	18
1987	4.8	0.20–45.8	7	19
1987	4.4	1.30–14.3	7	17
1988	2.26	0.10–17.30	4	15
1992	1.42	0.07–6.10	7	15
1992	0.26	0.06–5.04	3	20

**Table 2:** Asbestos exposure levels by process step in Korean asbestos textile industry (1987–1994)

Period	Content	Results (geometric mean, fiber/cc)	Measurement method
1987	Personal exposure assessment	Overall: 4.4 Fiberizing/Mixing: 4.5 Carding: 3.8 Spinning: 5.6 Twisting: 4.8 Weaving: 5.3	Phase Contrast Microscope
	Local concentration assessment	Overall: 5.7 Fiberizing/Mixing: 8.7 Carding: 6.6 Spinning: 6.6 Twisting: 5.2 Weaving: 5.5	
1988–1989	Personal exposure assessment	Fiberizing/Mixing: 0.23–3.69 Carding: 0.08–9.44 Spinning: 0.3–9.73 Twisting: 0.08–14.9 Weaving: 0.07–5.6	
1990	Personal exposure assessment	Carding: 0.38–17.3 Spinning: 0.26–15.0 Twisting: 0.1–12.6 Weaving: 0.28–17.2	
1992	Personal exposure assessment	Overall: 0.07–0.67	
1994	Personal exposure assessment	Mixing: 0.22–1.2 Carding: 0.23–9.24 Spinning: 0.41–8.93 Twisting: 0.21–9.83 Weaving: 2.61–11.58	
	Local concentration assessment	Mixing: 0.42 Carding: 0.61–10.93 Spinning: 0.78–3.65 Twisting: 1.01–9.66 Weaving: 3.18	

#### Environmental exposure

Based on the patient’s residential history, non-occupational exposure to asbestos was assessed through the method proposed by Magnani [36]. Given that she lived around 500–1000 m away from the asbestos textile factory for about 10 years from 1973 to 1982, the environmental exposure is applicable to high probability and middle intensity. In addition, considering that she brought her working clothes home and washed them at home, the domestic exposure is applicable to high probability and high intensity.

## Discussion and conclusion

According to a study on the occupational burden of asbestos-related diseases (ARD) in Korea, the number of ARD-attributable deaths and potential years of life lost (PYLL) due to all ARDs during 1998–2013 were 4492 and 71,763 respectively. The number of attributable death and PYLL due to ovarian cancer were 271, and 6331; additionally, the annual average PYLL (APYLL) and average age at death were 23.4 and 61.8. Ovarian cancer showed the highest APYLL among all ARDs because of the lowest age at death due to cancer and high life expectancy among women in Korea. The study showed that although the use of asbestos has ceased in Korea, the incidence of ARDs tends to increase [37].

Since asbestos is shaped like a long fiber, the macrophage is difficult to remove it when exposed to the body. Asbestos is not corroded by acid, alkali and is very durable that it remains in the body for a long time while chronically damaging the body [38, 39]. Asbestos fiber is basically found in all organs of the person exposed to asbestos [40]. According to the studies on translocation of inhaled asbestos textile, the asbestos inhaled by the respiratory organ remains for a long time within the lung tissue, causing chronic inflammation [39, 41]. Asbestos-induced lung inflammation reverses trans-mesothelial and trans-endothelial pressure gradients by increasing the pressure of lung interstitium and increases the permeability of asbestos. Asbestos textile is primarily pulled by the flow of pulmonary lymph from lung interstitium (primary translocation) to reach the bloodstream, and is continuously distributed to the whole body (secondary translocation). Since the translocation process of asbestos textile continues for decades, the latent period of asbestos-related disease is as long as 10 to 50 years [31, 34, 38, 39, 42, 43].

Since Cancers of the female reproductive system have multifactorial nature, it is important to conduct occupational studies that will gather detailed data on potential individual confounding factors. Studies on the mechanisms of carcinogenesis in the female reproductive organs are also needed in order to elucidate the possible role of chemical exposures in the development of these cancers [41]. She had four pregnancies, including two full-term deliveries and both of children had been breastfed for more than a year. In addition, risk factors such as hormone replacement therapy, family history of ovarian cancer and breast cancer and abnormality of BRCA1, 2 could not be confirmed, and use of talc powder on the perineal region, exposure to radiation, and smoking were not also found. In the analysis of her occupational and environmental exposure, the patient is presumed to be exposed to a large quantity of asbestos in the past. Moreover, the diagnosis of suspicious asbestosis with pleural plaques in chest CT also indicates that she has significant exposure to asbestos biologically. Since she was diagnosed with ovarian cancer pathologically, the possibility of misdiagnosis is low and the results of tumor marker of ovarian cancer (HE4) and ROMA (Risk of Ovarian Malignancy Algorithm) are also suggesting ovarian cancer rather than peritoneal mesothelioma.

Many previous studies showed that there was a positive correlation between cumulative quantity of exposure to asbestos and occurrence of asbestos-related disease [28–30]. According to the 2014 Helsinki criteria, in the case that cumulative quantity of exposure to asbestos was 25 fiber·year/cc or more, the relative risk of lung cancer doubled [44]. Since there has been no study on the dose-response relationship between exposure to asbestos and occurrence of ovarian cancer, the quantitative relationship cannot be identified but it can be presumed that there is a positive correlation even between asbestos and the occurrence of ovarian cancer. The level of the patient’s occupational exposure to asbestos is estimated at 19.25 or 53.6 fiber·year/cc or more when calculated based on the previous literature, and the exposure level at that time is confirmed to far exceed TWA 0.1 fiber/cc, which is PEL of Occupational Safety and Health Administration (OSHA) that is currently the international standard; ELT 1.0 fiber/cc; 0.1 fiber/cm3 in the case of asbestos of 5 μm or more in length, which is REL of National Institute for Occupational Safety and Health (NIOSH); and 0.1 fiber/cc, which is TLV of Association Advancing Occupational and Environmental Health (ACGIH). Given that levels of exposure to asbestos tend to decrease over time according to previous literature [14–17, 19], it is presumed that there would be a large quantity of exposure to asbestos in the 1970s when the patient worked at the factory. Considering the latent period of asbestos-related diseases is reported to be 10 to 50 years, we determined the ovarian cancer is highly correlated with the occupational exposure of asbestos and environmental exposure is a possible cause as well.

Considering that asbestos has a long latent period, it is expected that the effect of occupational and environmental exposure to asbestos on health will steadily continue in Korea [37]. In that sense, active monitoring and preventive surveillance are required along with clinical treatments for those exposed to asbestos in the past. In Korea, health damage due to environmental exposure to asbestos has relieved in accordance with the Asbestos Damage Relief Act in addition to the compensation for occupational exposure to asbestos under the Industrial Accident Compensation Insurance System. However, levels of compensation specified in the Asbestos Damage Relief Act are low compared to the Industrial Accident Compensation Insurance System. Provision of relief fund is limited only to primary malignant mesothelioma, primary lung cancer, asbestosis, and diffuse pleural thickening. Now that the causality between asbestos and ovarian cancer is confirmed to have sufficient evidence, the scope of compensation should actively cover all asbestos-related diseases including ovarian cancer.

## Abbreviations

BPLND: Bilateral pelvic lymph node dissection
BRCA1, 2: Breast cancer type 1, 2
BSO: Bilateral salpingo-oophorectomy
CA125: Cancer antigen 125
ELT: Excursion limit
HE4: Human epididymis protein4
PALND: Para-aortic lymph node dissection
PEL: Permissible exposure limit
REL: Recommended exposure limits
ROMA: Risk of Ovarian Malignancy Algorithm
TAH: Total abdominal hysterectomy
TLV: Threshold limit values
TWA: Time weighted average
